# Supplementary material for: Differential Effects of ABCG5/G8 Gene Region Variants on Lipid Profile, Blood Pressure Status, and Gallstone Disease History in Taiwan
Source: Genes (Basel). 2023 Mar 20;14(3):754. doi: 10.3390/genes14030754 (PMC10047937; doi:10.3390/genes14030754)
Supplement: Supplementary file 1 [file genes-14-00754-s001.zip › genes-2263057-supplementary.pdf]

Supplementary Method

Cardiometabolic traits analyzed

In addition to age, sex, body mass index and current smoking status and lipid profiles, the following clinical phenotypes were used in this study: waist circumference; waist–hip ratio; and systolic, mean, and diastolic blood pressure. Additionally, the following biochemical and hematological parameters were used: glucose metabolism parameters such as hemoglobin A1c (HbA1c) and fasting plasma glucose levels; liver and renal function parameters such as serum creatinine, blood urea nitrogen, aspartate aminotransferase (AST), alanine aminotransferase (ALT), γ-glutamyl transferase (γ-GT), albumin, total bilirubin, and uric acid levels; and platelet counts, white and red blood cell counts, and hemoglobin and hematocrit levels. The BMI and estimated glomerular filtration rate (eGFR) were calculated as reported previously [1]. Because of the absence of data related to urine creatinine levels, only spot urine albumin levels were used to evaluate albuminuria.

Definitions of metabolic syndrome

Because medication histories were unavailable, metabolic syndrome characteristics were based on the recent update of the third report of the National Cholesterol Education Program's Adult Treatment Panel III criteria [2] with modifications. Participants with three or more of the following attributes are typically defined as having metabolic syndrome: (1) BP of ≥130/85 mmHg or a history of hypertension; (2) triglyceride level of ≥150 mg/dL; (3) high-density lipoprotein cholesterol level of <40 mg/dL for men or <50 mg/dL for women; (4) fasting plasma glucose of ≥100 mg/dL or a history of DM; and (5) waist circumference of >90 cm for men or>80 cm for women.

Reference:

1. Hsu LA, Chou HH, Teng MS, Wu S, Ko YL. Circulating chemerin levels are determined through circulating platelet counts in nondiabetic Taiwanese people: A bidirectional Mendelian randomization study. Atherosclerosis. 2021 Mar;320:61-69.

2. Grundy SM, Cleeman JI, Daniels SR, Donato KA, Eckel RH, Franklin BA, et al. Diagnosis and management of the metabolic syndrome: an American Heart Association/National Heart, Lung, and Blood Institute Scientific Statement. Circulation. 2005; 11: 2735–52.

Table S1. Selected *ABCG5,* and *ABCG8* variants in Taiwan Biobank participants: Data derived from the whole genome sequence and Axiom Genome-Wide CHB Array plates with genotype imputation

Ref	Gene	SNPs	Position (GRCh37)	Ref/Alt	Intron or Exon number	Exonic Function	mRNA sequence	AA Change.	HWE	MAF#					SNP function	RegulomeDB	
									TWB	TWB	East Asian	Europe	American	African		Rank	Score
0	*ABCG5*	rs374012053	44040355	C/G	13	Nonsynonymous	c.G1856C	p.R619T	1	0.00067	0.00100	0	0	0		3a	0.3298
14	*ABCG5*	rs6720173	44040401	G/C	13	Nonsynonymous	c.C1810G	p.Q604E	0.6763	0.10370	0.12100	0.16400	0.30100	0.32980	Splicing (ESE or ESS)	3a	0.7752
0	*ABCG5*	rs200839584	44047133	C/T	11	Nonsynonymous	c.G1570A	p.V524I	1	0.00067	0.00100	0	0.00100	0		2c	1.0000
0	*ABCG5*	rs199984328*	44047175	G/T	11	Nonsynonymous	c.C1528A	p.H510N	0.1570	0.00700	0.00400	0	0	0		2b	0.7384
0	*ABCG5*	rs139361486	44050052	G/A	10	Synonymous	c.C1347T	p.S449S	1	0.00602	0.00400	0	0	0		5	0.2860
0	*ABCG5*	rs536081800*	44050062	C/T	10	Nonsynonymous	c.G1337A	p.R446Q	1	0.00040	0.00100	0	0	0		5	0.1345
8	*ABCG5*	rs199689137	44050063	G/A	10	Nonsense	c.C1336T	p.R446X	1	0.00033	0.00190[a]	0.00005[a]	0[a]	0.00012[a]		5	0.1345
0	*ABCG5*	rs200597050	44050073	A/G	9	Synonymous	c.T1326C	p.F442F	1	0.00067	0.00100	0	0	0		5	0.4161
0	*ABCG5*	rs575266356	44051065	G/C	9	Nonsynonymous	c.C1311G	p.N437K	1	0.00067	0	0	0	0		5	0.6312
0	*ABCG5*	rs143680435	44051153	C/T	9	Nonsynonymous	c.G1223A	p.R408Q	1	0.00033	0.00100	0	0	0		5	0.1345
1	*ABCG5*	rs119480069*	44051210	C/T	8	Nonsynonymous	c.G1166A	p.R389H	1	0.00310	0.00100	0	0	0		5	0.1345
0	*ABCG5*	rs745606275	44051408	G/C	8	Nonsynonymous	c.C1068G	p.F356L	1	0.00033	0.00030[a]	0[a]	0[a]	0[a]		7	0.1841
0	*ABCG5*	rs775348848	44051409	A/T	8	Nonsynonymous	c.T1067A	p.F356Y	1	0.00067	0.00030[a]	0[a]	0[a]	0[a]		7	0.1841
0	*ABCG5*	rs748009304	44052032	G/C	7	Nonsynonymous	c.C900G	p.F300L	1	0.00067	0.00100[a]	0[a]	0[a]	0[a]		5	0.9583
0	*ABCG5*	rs373182673	44052098	C/T	7	Synonymous	c.G834A	p.A278A	1	0.00067	0[a]	0.00004[a]	0[a]	0[a]		4	0.6091
0	*ABCG5*	rs566982836	44052099	G/A	7	Nonsynonymous	c.C833T	p.A278V	1	0.00067	0.00100	0	0	0		4	0.6091
0	*ABCG5*	rs539178581	44052104	C/T	7	Synonymous	c.G828A	p.T276T	1	0.00067	0	0	0.00100	0		4	0.6091
0	*ABCG5*	rs148186696*	44053537	C/T	6	Nonsynonymous	c.G758A	p.R253H	1	0.00040	0.00030[a]	0.00009[a]	0[a]	0.00005[a]		5	0.1345
0	*ABCG5*	rs140111105	44053544	G/A	6	Nonsense	c.C751T	p.Q251X	1	0.00033	0.00018[b]	0[b]	0[b]	0.00006[b]		5	0.1345
0	*ABCG5*	rs775594151	44055147	G/A	5	Synonymous	c.C609T	p.I203I	1	0.00201	0[a]	0.00004[a]	0[a]	0[a]		5	0.1345
0	*ABCG5*	rs780730956	44055172	G/A	5	Nonsynonymous	c.C584T	p.T195M	1	0.00033	0[a]	0.00001[a]	0[a]	0[a]		5	0.1345
0	*ABCG5*	rs767751451*	44055213	T/G	5	Synonymous	c.A543C	p.A181A	1	0.00050	0.00100[a]	0.00001[a]	0[a]	0[a]		5	0.3248
0	*ABCG5*	rs748096191*	44058971	C/G	4	Nonsynonymous	c.G438C	p.E146D	1	0.00060	0[a]	0[a]	0[a]	0.00002[a]		4	0.6091
17	*ABCG5*	rs6756629*	44065090	G /A	2	Nonsynonymous	c.C148T	p.R50C	0.6410	0.01500	0.01390	0.07550	0.09900	0.10290	Splicing (ESE or ESS)	5	0.1345
0	*ABCG5*	rs72542426	44065680	C/G	1	Nonsynonymous	c.G139C	p.V47L	1	0.00067	0	0	0.00300	0.01440		4	0.6091
0	*ABCG5*	rs781098379	44065755	G/A	1	Nonsense	c.C64T	p.Q22X	1	0.00067	0.00010[b]	0.00004[b]	0[b]	0[b]		4	0.6091
0	*ABCG5*	rs200471843	44065786	C/A	1	Synonymous	c.G33T	p.G11G	1	0.00335	0.00100	0	0	0		4	0.6091

0	*ABCG5*	rs72542428	44065792	G/A	1	Synonymous	c.C27T	p.P9P	0.1636	0.01104	0.00890	0	0.00100	0		2a	0.6541
0	*ABCG5*	rs560839317*	44065894	G/A	5’UTR	--	--	--	1	0.00130	0.00100	0	0	0		2b	0.5881
0	*ABCG5*	rs189132480*	44065924	C/T	5’UTR	--	--	--	1	0.00100	0.00200	0	0	0		2b	0.6958
0	*ABCG5;ABCG8*	rs138510485	44065996	C/T	upstream	--	--	--	1	0.00033	0.00200	0	0	0		4	0.6091
0	*ABCG5;ABCG8*	rs913853612	44066072	G/A	upstream	--	--	--	1	0.00033	0[a]	0.00005[a]	0[a]	0.00012[a]		4	0.6091
6	*ABCG8*	rs3806471	44066174	T/G	5’UTR	--	--	--	0.5314	0.14460	0.84230	0.29720	0.35600	0.39790	TFBS	4	0.6091
0	*ABCG8*	rs200951677	44066207	G/A	1	Synonymous	c.G15A	p.A5A	1	0.00033	0.00100	0	0	0		4	0.6091
32	*ABCG8*	rs11887534*	44066247	G/C	1	Nonsynonymous	c.G55C	p.D19H	0.7100	0.01510	0.01390	0.07950	0.09700	0.07640	Splicing (ESE or ESS)	4	0.6091
11	*ABCG8*	rs4148211	44071743	A/G	2	Nonsynonymous	c.A161G	p.Y54C	0.6276	0.12150	0.85120	0.37080	0.35000	0.12930	Splicing (ESE or ESS)	4	0.6091
0	*ABCG8*	rs754458742	44078728	G/A	4	Nonsynonymous	c.G328A	p.G110R	1	0.00033	0.00004[b]	0[b]	0[b]	0[b]		5	0.1345
0	*ABCG8*	rs968485818	44078789	G/T	4	Nonsynonymous	c.G389T	p.G130V	1	0.00033	0[c]	0[c]	0[c]	0[c]		5	0.0523
0	*ABCG8*	rs756189400	44078829	G/A	4	Synonymous	c.G429A	p.L143L	1	0.00033	0.00008[b]	0[b]	0[b]	0[b]		5	0.5800
0	*ABCG8*	rs56132765*	44078853	G/A	4	Synonymous	c.G453A	p.V151V	0.7820	0.01530	0.01390	0.07260	0.09200	0.09460		5	0.0000
0	*ABCG8*	rs750352877*	44078879	A/G	4	Nonsynonymous	c.A479G	p.N160S	1	0.00070	0[a]	0.00001[a]	0.00007[a]	0[a]		5	0.0231
0	*ABCG8*	rs762440574	44078921	G/A	4	Nonsynonymous	c.G521A	p.R174Q	1	0.00033	0[a]	0[a]	0.00015[a]	0[a]		5	0.1345
0	*ABCG8*	rs201121311	44079502	G/A	5	Nonsynonymous	c.G571A	p.V191M	1	0.00033	0.00100	0	0	0		4	0.6091
0	*ABCG8*	rs9282574	44079559	G/A	5	Nonsynonymous	c.G628A	p.V210M	1	0.00201	0	0	0.00700	0.06510	Splicing (ESE or ESS)	3a	0.8507
0	*ABCG8*	rs267599381	44079620	A/G	5	Nonsynonymous	c.A689G	p.N230S	1	0.00033	0.00002[b]	0[b]	0[b]	0[b]		4	0.6091
0	*ABCG8*	rs199737442	44079829	C/A	6	Nonsynonymous	c.C786A	p.N262K	1	0.00100	0.00100	0	0	0		4	0.6091
1	*ABCG8*	rs137852990	44079831	G/A	6	Nonsynonymous	c.G788A	p.R263Q	1	0.00100	0.00100	0.00100	0	0		4	0.6091
0	*ABCG8*	rs775468682	44079861	G/A	6	Nonsynonymous	c.G818A	p.R273H	1	0.00033	0.00004[b]	0.00002[b]	0.00006[b]	0[b]		4	0.6091
0	*ABCG8*	rs117221284	44079913	C/T	6	Synonymous	c.C870T	p.T290T	1	0.00770	0.00600	0	0	0		5	0.1550
0	*ABCG8*	rs745365455	44079981	G/A	6	Nonsynonymous	c.G938A	p.R313H	1	0.00033	0.00002[b]	0.00002 [b]	0[b]	0[b]		5	0.1345

Chr: chromosome, SNP: single nucleotide polymorphism, Ref/Alt: reference/alternate allele, HWE: Hardy-Weinberg equilibrium, MAF: minor allele frequency, mRNA: messenger RNA, AA: amino acid, 5’UTR: 5’ untranslated region, ESE or ESS: exon splicing enhancer or exon splicing silencer, TFBS: transcriptional factor binding site.

*Single nucleotide polymorphism available on the Axiom Genome-Wide CHB Array plates with genotype imputation of Taiwan biobank (TWB) data.

HWE and MAF (TWB) data were derived from Axiom Genome-Wide CHB Array plates with genotype imputation of Taiwan biobank (TWB), or if not available, from whole genome sequence of TWB data.

MAF (except for TWB) was mainly accessed from 1000 Genomes Project and if the data from 1000 Genomes Project was not available, gnomAD-Genomes (label as “a”), gnomAD–Exomes (label as ‘b’), and dbGaP Population Frequency (label as ‘c’) on the PUBMed website were used.

SNP function information was obtained from SNPinfo Web Server.

RegulomeDB Rank and Score information was obtained from RegulomeDB database. The lower rank indicates the more evidences that the SNP may be located in a functional region and the scores, ranging from 0 to 1, which was closer to 1, the more possibility that the SNP would play a regulatory role.

Table S2. Lead single nucleotide polymorphisms (SNPs) for various phenotypes at the *ABCG5, ABCG8* and *PLEKHH2* gene region: Data derived from the Axiom Genome-Wide CHB Array plates with genotype imputation

Chr	Gene	Associated phenotypes	SNPs	Position (GRCh37)	Ref/Alt	Intron or Exon number	Exonic Function	mRNA sequence	AA Change.	HWE	MAF#					SNP function	RegulomeDB	
										TWB	TWB	East Asian	Europe	American	African		Rank	Score
2	*PLEKHH2*	Total, LDL and non-HDL cholesterol levels	rs75832441	43965298	G/A	Intron	--	--	--	0.6150	0.01040	0.00800	0.02600	0.05300	0.01880	--	5	0.1345
2	*PLEKHH2*	Mean blood pressure	rs7596913	43975512	G/C	Intron	--	--	--	0.8851	0.19600	0.17660	0.28330	0.45100	0.81620	--	5	0.8994
2	*PLEKHH2*	Diastolic blood pressure	rs2060173	43975957	A/G	Intron	--	--	--	0.8500	0.19700	0.17760	0.28330	0.45100	0.81540	--	5	0.5896
2	*ABCG5*	Gallstone disease history	rs115445558	44063098	G/T	Intron	--	--	--	0.1980	0.01540	0.07950	0.09900	0.10290	0.01690	--	6	0.1865

Abbreviation as in Supplementary Table 1.±

MAF (except for TWB) was accessed from 1000 Genomes Project on the PUBMed website.

SNP function information was obtained from SNPinfo Web Server.

RegulomeDB Rank and Score information was obtained from RegulomeDB database. The lower rank indicates the more evidences that the SNP may be located in a functional region and the scores, ranging from 0 to 1, which was closer to 1, the more possibility that the SNP would play a regulatory role.

Table S3. Phenotype–genotype associations of *ABCG5* and *ABCG8* exonic mutations and promoter variants with no significant association with lipid profile or gallstone disease history

Genetic variants	Genotypes			*p1* value*	Beta	SE	*p2* value*
ABCG5 rs536081800 (108,518)	CC (108,427)	CT (91)	TT (0)				
Total cholesterol# (mg/dL)	193.0 (171.0 - 217.0)	205.0 (174.0 - 228.0)	--	0.0257	0.0164	0.0082	0.0458
LDL cholesterol# (mg/dL)	119.0 (99.0 - 141.0)	127.0 (101.0 - 148.0)	--	0.2069	0.0167	0.0120	0.1640
Non-HDL cholesterol# (mg/dL)	138.0 (116.0 - 162.0)	146.0 (121.0 - 171.0)	--	0.0707	0.0226	0.0108	0.0369
HDL cholesterol# (mg/dL)	53.0 (45.0 - 63.0)	54.0 (47.0 - 66.0)	--	0.3073	0.0048	0.0097	0.6245
Triglyceride# (mg/dL)	91.0 (64.0 - 133.0)	90.0 (58.0 - 133.0)	--	0.6128	0.0234	0.0227	0.3035
Remnant cholesterol# (mg/dL)	16.0 (11.5 - 23.0)	17.0 (10.0 - 26.0)	--	0.6501	0.0036	0.0283	0.8986
Gallstone disease (%)	4.55% (5332)	7.22% (7)	--	0.2079	0.5202	0.3969	0.1899
Family history of gallstone disease (%)	7.53% (8828)	7.22% (7)	--	0.9060	-0.0554	0.3926	0.8877
ABCG5 rs148186696 (108,866)	CC (108,775)	CT (91)	TT (0)				
Total cholesterol# (mg/dL)	193.0 (171.0 - 217.0)	202.0 (175.0 - 219.0)	--	0.0593	0.0125	0.0082	0.1284
LDL cholesterol# (mg/dL)	119.0 (99.0 - 141.0)	123.0 (108.0 – 144.0)	--	0.0443	0.0263	0.0120	0.0281
Non-HDL cholesterol# (mg/dL)	138.0 (116.0 - 162.0)	145.0 (123.0 - 168.0)	--	0.0784	0.0225	0.0108	0.0375
HDL cholesterol# (mg/dL)	53.0 (45.0 - 63.0)	52.0 (44.0 - 60.0)	--	0.8802	0.0046	0.0097	0.6356
Triglyceride# (mg/dL)	91.0 (64.0 - 133.0)	93.0 (61.0 - 138.0)	--	0.7608	0.0066	0.0227	0.7707
Remnant cholesterol# (mg/dL)	16.0 (11.0 - 23.0)	16.0 (13.0 - 24.0)	--	0.6214	0.0128	0.0283	0.6515
Gallstone disease (%)	4.56% (5356)	3.00% (3)	--	0.4557	-0.3797	0.5897	0.5197
Family history of gallstone disease (%)	7.54% (8860)	6.00% (6)	--	0.5607	-0.2548	0.4213	0.5453
ABCG5 rs767751451 (108,226)	TT (108,123)	TG (103)	GG (0)				
Total cholesterol# (mg/dL)	193.0 (171.0 - 217.0)	196.0 (172.0 - 216.0)	--	0.6973	-0.0009	0.0077	0.9105
LDL cholesterol# (mg/dL)	119.0 (99.0 - 141.0)	121.0 (100.0 - 141.0)	--	0.6731	0.0007	0.0113	0.9487
Non-HDL cholesterol# (mg/dL)	138.0 (116.0 - 162.0)	139.0 (118.0 - 162.0)	--	0.9362	-0.0056	0.0102	0.5825
HDL cholesterol# (mg/dL)	53.0 (45.0 - 63.0)	56.0 (47.0 - 65.0)	--	0.1860	0.0148	0.0091	0.1052
Triglyceride# (mg/dL)	91.0 (64.0 - 133.0)	87.0 (65.0 - 118.0)	--	0.3885	-0.0289	0.0213	0.1752
Remnant cholesterol# (mg/dL)	16.0 (11.0 - 23.0)	16.0 (11.0 - 22.0)	--	0.4274	-0.0276	0.0266	0.3004
Gallstone disease (%)	4.56% (5328)	5.71% (6)	--	0.5705	0.2149	0.4234	0.6117
Family history of gallstone disease (%)	7.55% (8818)	5.71% (6)	--	0.4777	-0.3002	0.4206	0.4755
ABCG5 rs189132480 (108,607)	CC (108,375)	CT (232)	TT (0)				
Total cholesterol# (mg/dL)	193.0 (171.0 - 217.0)	196.0 (175.0 - 215.0)	--	0.4260	0.0019	0.0051	0.7054
LDL cholesterol# (mg/dL)	119.0 (99.0 - 141.0)	121.0 (99.3 - 140.0)	--	0.6997	0.0012	0.0075	0.8691
Non-HDL cholesterol# (mg/dL)	138.0 (116.0 - 162.0)	139.0 (117.0 - 159.8)	--	0.7454	0.00003	0.0068	0.9966
HDL cholesterol# (mg/dL)	53.0 (45.0 - 63.0)	56.0 (46.3 - 64.0)	--	0.1462	0.0122	0.0060	0.0429
Triglyceride# (mg/dL)	91.0 (64.0 - 133.0)	91.0 (66.0 - 127.8)	--	0.8801	-0.0108	0.0141	0.4454
Remnant cholesterol# (mg/dL)	16.0 (11.0 - 23.0)	16.0 (11.0 - 22.0)	--	0.6702	-0.0032	0.0177	0.8551
Gallstone disease (%)	4.56% (5336)	6.10% (15)	--	0.2468	0.2859	0.2690	0.2879
Family history of gallstone disease (%)	7.54% (8829)	8.54% (21)	--	0.5533	0.1379	0.2285	0.5463
ABCG8 rs750352877 (108,404)	AA (108,260)	AG (144)	GG (0)				
Total cholesterol# (mg/dL)	193.0 (171.0 - 217.0)	196.5 (167.3 - 216.0)	--	0.7794	-0.0009	0.0064	0.8946
LDL cholesterol# (mg/dL)	119.0 (99.0 - 141.0)	115.5 (99.0 - 141.0)	--	0.5562	-0.0073	0.0095	0.4410
Non-HDL cholesterol# (mg/dL)	138.0 (116.0 - 162.0)	137.0 (113.0 - 162.8)	--	0.5662	-0.0072	0.0086	0.4020
HDL cholesterol# (mg/dL)	53.0 (45.0 - 63.0)	55.0 (45.0 - 63.8)	--	0.6889	-0.0012	0.0076	0.8709
Triglyceride# (mg/dL)	91.0 (64.0 - 133.0)	87.0 (66.0 - 126.5)	--	0.7174	-0.0064	0.0178	0.7178
Remnant cholesterol# (mg/dL)	16.0 (11.0 - 23.0)	17.0 (11.3 - 25.0)	--	0.4734	0.0151	0.0225	0.5018
Gallstone disease (%)	4.56% (5326)	3.07% (5)	--	0.3634	-0.4899	0.4564	0.2831
Family history of gallstone disease (%)	7.54% (8824)	6.75% (11)	--	0.7015	-0.1247	0.3125	0.6899

Data presented as median (interquartile range).
Abbreviation as in Table 1.
Number of the participants shown in brackets after the genotypes.
Participants were analyzed after the exclusion of those with a history of hyperlipidemia.
**p1* value: unadjusted; *p2* value: adjusted for age, sex, BMI, and current smoking.

Table S4. The multicollinearity between the lipid levels in the stepwise multiple linear regression model. The *ABCG5* rs75832441 has the lowest tolerance (0.319, that is <0.4) for all three phenotypes analyzed. Thus, we removed *ABCG5* rs7583244 (since lowest tolerance) from the model in Table 3.

	Unstandardised Coefficients		Standardised Coefficients	T	Sig.	Collinearity Statistics	
Model	B	Std. Error	Beta			Tolerance	VIF
Total cholesterol level							
(Constant)	2.168	.002		1000.319	0.000		
AGE	.001	.000	.167	58.250	0.000	1.000	1.000
SEX	.015	.001	.090	27.298	.000	.755	1.325
BMI	.001	.000	.061	20.618	.000	.946	1.057
SMK	.000	.001	-.002	-.682	.495	.780	1.283
ABCG5 rs119480069	.025	.003	.025	8.627	.000	1.000	1.000
ABCG5 rs199984328	.008	.003	.012	2.273	.023	.319	3.133
ABCG5 rs560839317	.019	.004	.012	4.178	.000	1.000	1.000
ABCG5 rs748096191	.024	.007	.010	3.503	.000	1.000	1.000
ABCG5 rs75832441	.004	.003	.008	1.496	.135	.319	3.133
LDL cholesterol level							
(Constant)	1.904	.003		585.093	0.000		
AGE	.001	.000	.109	37.728	0.000	1.000	1.000
SEX	.001	.001	.003	.935	.350	.755	1.325
BMI	.004	.000	.137	46.313	0.000	.946	1.057
SMK	-.005	.001	-.016	-4.837	.000	.780	1.283
ABCG5 rs119480069	.039	.004	.026	8.909	.000	1.000	1.000
ABCG5 rs199984328	.016	.005	.016	3.069	.002	.319	3.133
ABCG5 rs560839317	.023	.007	.010	3.524	.000	1.000	1.000
ABCG5 rs748096191	.036	.010	.010	3.552	.000	1.000	1.000
ABCG5 rs75832441	.004	.004	.004	.832	.405	.319	3.133
Non-HDL cholesterol level							
(Constant)	1.846	.003		725.514	0.000		
AGE	9.470E-05	.000	.010	3.860	.000	1.000	1.000
SEX	.063	.001	.286	98.218	0.000	.755	1.325
BMI	-.009	.000	-.337	-129.740	0.000	.946	1.057
SMK	-.011	.001	-.042	-14.568	.000	.780	1.283
ABCG5 rs119480069	.000	.003	.000	-.125	.901	1.000	1.000
ABCG5 rs199984328	-.003	.004	-.003	-.705	.481	.319	3.133
ABCG5 rs560839317	-.012	.005	-.006	-2.216	.027	1.000	1.000
ABCG5 rs748096191	.001	.008	.000	.108	.914	1.000	1.000
ABCG5 rs75832441	.000	.003	.001	.142	.887	.319	3.133

Table S5. Subgroup analysis between rs11887534 and gallstone disease history: age and sex categories

Gallstone disease history (%)	GG	GC	CC	*p1* value	Beta	SE	*p2* value	interaction *P*
Male	4.70% (1939)	6.98% (84)	20.00% (2)	9.80×10^{-5}	0.4435	0.1126	8.10×10^{-5}	0.3960
Female	4.38% (3194)	6.01% (139)	4.76% (1)	0.0009	0.3088	0.0883	0.0005	
Young age (< 50 y/o)	2.72% (1470)	3.85% (62)	11.11% (1)	0.0076	0.3767	0.1301	0.0038	0.8474
Old age (□ 50 y/o)	6.10% (3663)	8.46% (161)	9.09% (2)	0.0001	0.3536	0.0821	1.60×10^{-5}	

Data presented as percentage (number).

p1 value: unadjusted; *p2* value for male and female: adjusted for age, BMI, and current smoking. *p2* value for young and old age: adjusted for sex, BMI, and current smoking.

Table S6. Genotype-phenotype association analysis of *ABCG5* and *ABCG8* lead single nucleotide variants for lipid profile and gallstone disease history

Genetic variants	Genotypes			*p1* value*	Beta	SE	*p2* value*
PLEKHH2 rs75832441 (117,601)	GG (115,206)	GA (2384)	AA (11)				
Total cholesterol# (mg/dL)	193.0 (171.0 - 217.0)	198.0 (174.0 - 223.0)	197.0 (186.3 - 20830)	3.41×10^{-7}	0.0093	0.0016	1.20×10^{-8}
LDL cholesterol# (mg/dL)	119.0 (99.0 - 140.0)	123.0 (103.0 - 146.0)	130.5 (107.8 - 134.0)	3.67×10^{-7}	0.0137	0.0024	2.20×10^{-8}
Non-HDL cholesterol# (mg/dL)	138.0 (116.0 - 162.0)	142.0 (119.0 - 167.0)	147.5 (131.8 - 165.3)	1.34×10^{-7}	0.0132	0.0022	2.30×10^{-9}
HDL cholesterol# (mg/dL)	53.0 (45.0 - 63.0)	53.0 (45.0 - 63.0)	51.0 (41.8 - 59.0)	0.7663	-0.0008	0.0020	0.6819
Triglyceride# (mg/dL)	91.0 (64.0 - 133.0)	92.0 (63.0 - 137.0)	98.5 (66.3 - 148.0)	0.3389	0.0083	0.0046	0.0704
Remnant cholesterol# (mg/dL)	19.0 (14.0 - 26.0)	16.0 (11.0 - 23.0)	19.5 (14.5 - 31.3)	0.0788	0.0127	0.0056	0.0229
Gallstone disease (%)	4.57% (5259)	4.07% (97)	18.18% (2)	0.0493	-0.0845	0.1027	0.4107
Family history of gallstone disease (%)	7.54% (8692)	7.17% (171)	0.00% (0)	0.5065	-0.0619	0.0798	0.4381
ABCG5-ABCG8 rs115445558 (117,578)	GG (114,014)	GC (3532)	CC (32)				
Total cholesterol# (mg/dL)	193.0 (171.0 - 217.0)	193.0 (170.0 - 217.0)	206.5 (191.5 - 229.3)	0.2245	-0.0007	0.0013	0.5970
LDL cholesterol# (mg/dL)	119.0 (99.0 - 141.0)	119.0 (99.0 - 141.0)	129.5 (110.0 - 145.3)	0.3121	-0.0016	0.0020	0.4351
Non-HDL cholesterol# (mg/dL)	138.0 (116.0 - 162.0)	137.0 (115.0 - 161.0)	151.0 (137.5 - 171.3)	0.0996	-0.0009	0.0018	0.5977
HDL cholesterol# (mg/dL)	53.0 (45.0 - 63.0)	54.0 (45.0 - 63.0)	54.0 (45.8 - 62.0)	0.6031	-0.0002	0.0016	0.9093
Triglyceride# (mg/dL)	91.0 (64.0 - 133.0)	90.0 (63.0 - 133.0)	99.0 (76.8 - 166.0)	0.2170	0.0025	0.0037	0.5039
Remnant cholesterol# (mg/dL)	16.0 (11.0 - 23.0)	16.0 (12.0 - 23.0)	21.0 (13.0 - 34.0)	0.0756	0.0038	0.0045	0.4059
Gallstone disease (%)	4.50% (5126)	6.37% (225)	9.38% (3)	4.12×10^{-7}	0.3666	0.0691	1.14×10^{-7}
Family history of gallstone disease (%)	7.50% (8549)	8.78% (310)	9.38% (3)	0.0166	0.1685	0.0594	0.0045

Data presented as median (interquartile range).
Abbreviation as in Table 1.
Number of the participants shown in brackets after the genotypes.
Participants were analyzed after the exclusion of those with a history of hyperlipidemia.
**p1* value: unadjusted; *p2* value: adjusted for age, sex, BMI, and current smoking.

Table S7. Baseline characteristics of Taiwan Biobank participants

Clinical and laboratory parameters	Participants with GWAS data (n = 117,679)
Blood Pressure	
Systolic BP† (mmHg)	115.5 (105.3 - 127.0)
Diastolic BP† (mmHg)	71.0 (65.0 - 79.0)
Mean BP† (mmHg)	86.2 (78.8 - 94.6)
Glucose metabolism	
Fasting plasma glucose†† (mg/dL)	92.0 (87.0 - 97.0)
HbA1C†† (%)	5.6 (5.4 - 5.8)
Insulin resistance surrogate markers	
TyG index#, ††	8277.0 (5696.0 - 12500.0)
TyG-BMI#, †† ($\times 10^3$)	197.29 (127.32 – 316.91)
TyG-WC#, †† ($\times 10^3$)	683.52 (444.73 – 1080.97)
Uric acid	
Uric acid††† (mg/dL)	5.2 (4.4 - 6.2)
Renal function	
Creatinine (mg/dL)	0.68 (0.58 - 0.84)
eGFR (mL/min/1.73 m^2)	100.16 (86.88 - 115.56)
Albuminuria (mg/L)	8.7 (5.3 - 15.2)
Liver function	
AST (U/L)	23.0 (20.0 - 27.0)
ALT (U/L)	19.0 (14.0 - 27.0)
γGT (U/L)	17.0 (12.0 - 26.0)
Serum albumin (g/dL)	4.5 (4.4 - 4.7)
Total bilirubin (mg/dL)	0.6 (0.5 - 0.8)
Hematological parameters	
Leukocyte count (10^3/μL)	5.6 (4.7 - 6.7)
Hematocrit (%)	41.5 (38.9 - 44.5)
Platelet count (10^3/μL)	238.0 (202.0 - 277.0)
Red blood cell count (10^6/μL)	4.7 (4.4 - 5.1)
Hemoglobin (g/dL)	13.7 (12.8 - 14.8)
Atherosclerotic risk factors	
Diabetes mellitus (%)	9.47% (11142)
Hypertension (%)	22.27% (26212)
Current smoking (%)	19.67% (23,148)
Gout (%)	3.86% (4548)
Microalbuminuria (%)	11.19% (13141)
Metabolic syndrome (%)	25.52% (30,035)

GWAS: genome-wide association study, BP: blood pressure, HbA1C: hemoglobin A1C, TyG: triglyceride glucose, BMI: body mass index, WC: waist circumference, eGFR: estimated glomerular filtration rate, AST: aspartate aminotransferase, ALT: alanine aminotransferase, γGT: γ-Glutamyl transferase.

Participants were analyzed after the exclusion of those with a history of †hypertension, ††diabetes mellitus, †††gout, and #hyperlipidemia.

Level was presented as median (interquartile range) or percentage (number).

Figure S1. Regional association analysis of *ABCG5* and *ABCG8* loci variants and lipid profile

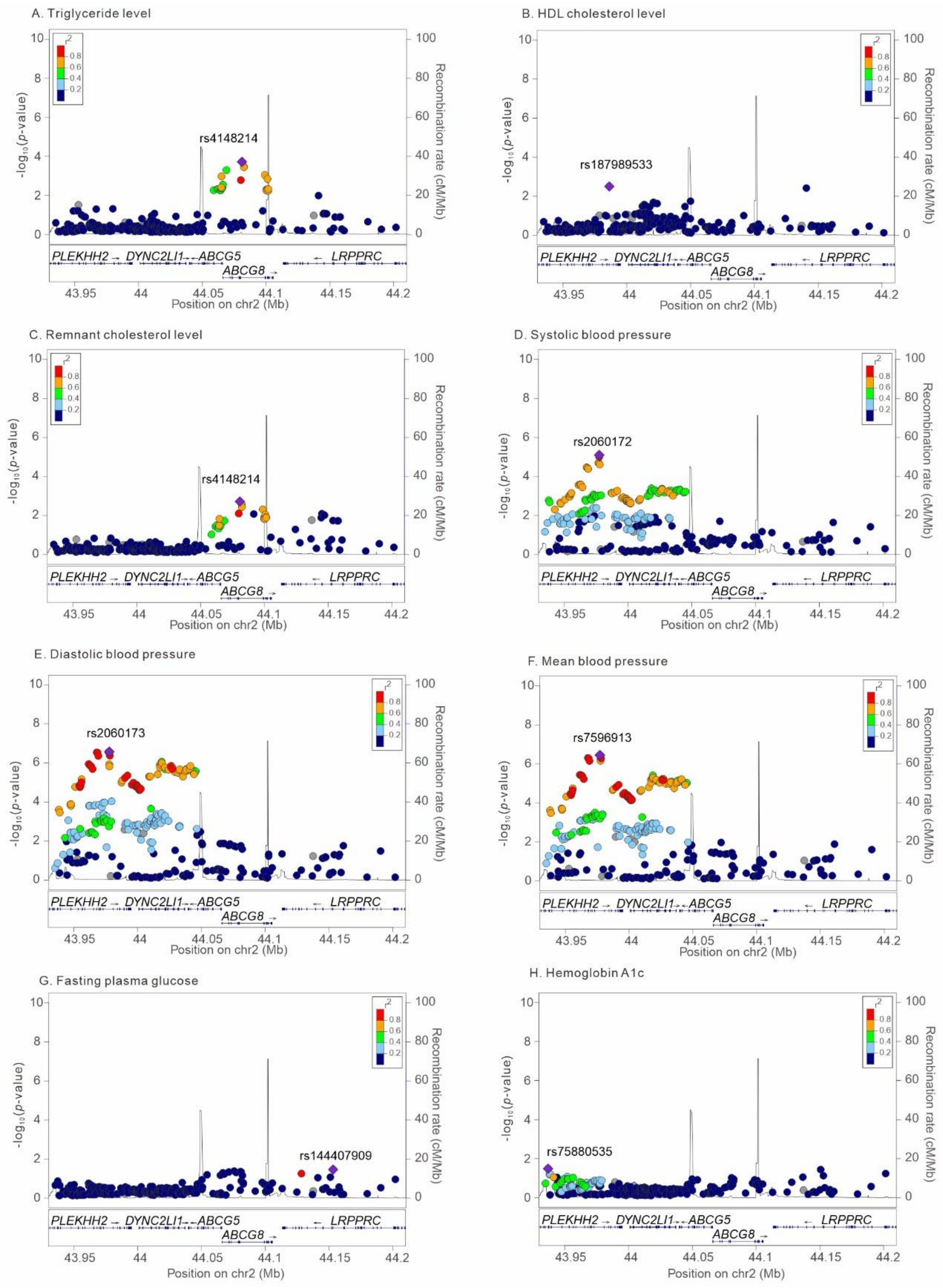

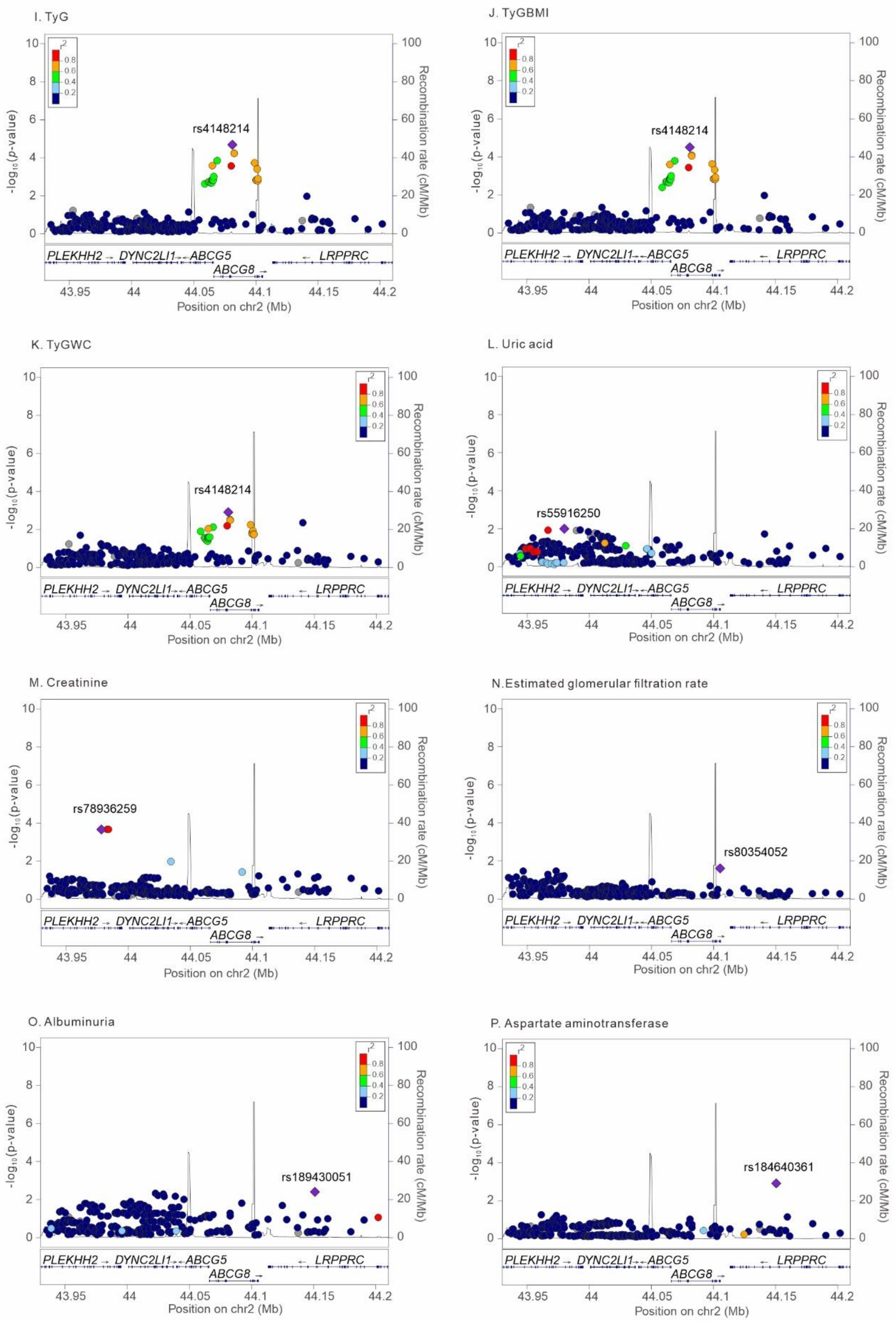
I. TyG
rs4148214
J. TyGBMI
rs4148214
K. TyGWC
rs4148214
L. Uric acid
rs55916250
M. Creatinine
rs78936259
N.Estimated glomerular filtration rate
rs80354052
O. Albuminuria
rs189430051
P. Aspartate aminotransferase
rs184640361
-log10(p-value)
Recombination rate (cM/Mb)
Position on chr2 (Mb)
PLEKHH2
DYNC2LI1
ABCG5
ABCG8
LRPPRC

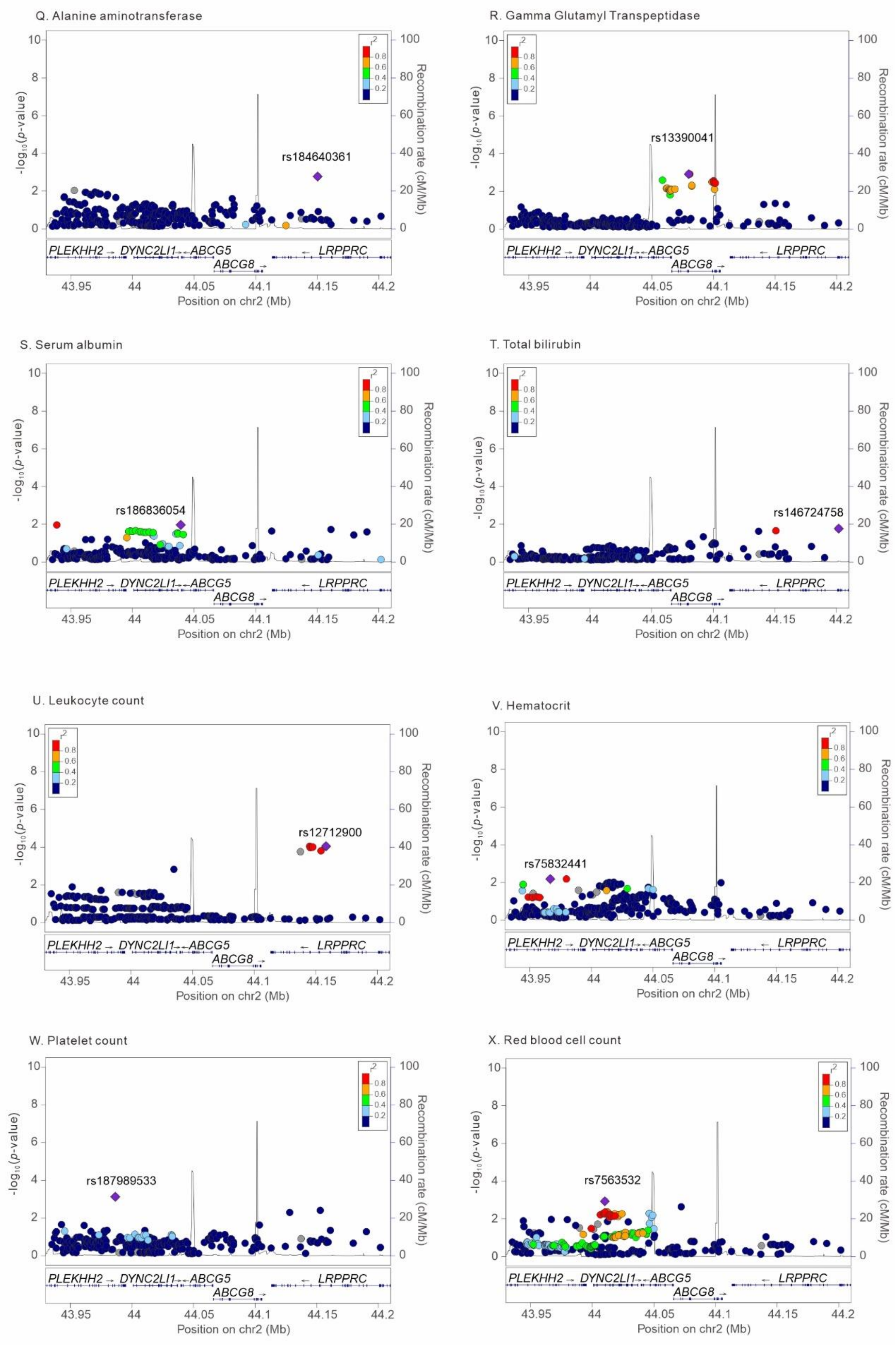
Q. Alanine aminotransferase
rs184640361
R. Gamma Glutamyl Transpeptidase
rs13390041
S. Serum albumin
rs186836054
T. Total bilirubin
rs146724758
U. Leukocyte count
rs12712900
V. Hematocrit
rs75832441
W. Platelet count
rs187989533
X. Red blood cell count
rs7563532
-log10(p-value)
Recombination rate (cM/Mb)
Position on chr2 (Mb)
PLEKHH2
DYNC2LI1
ABCG5
ABCG8
LRPPRC
r2

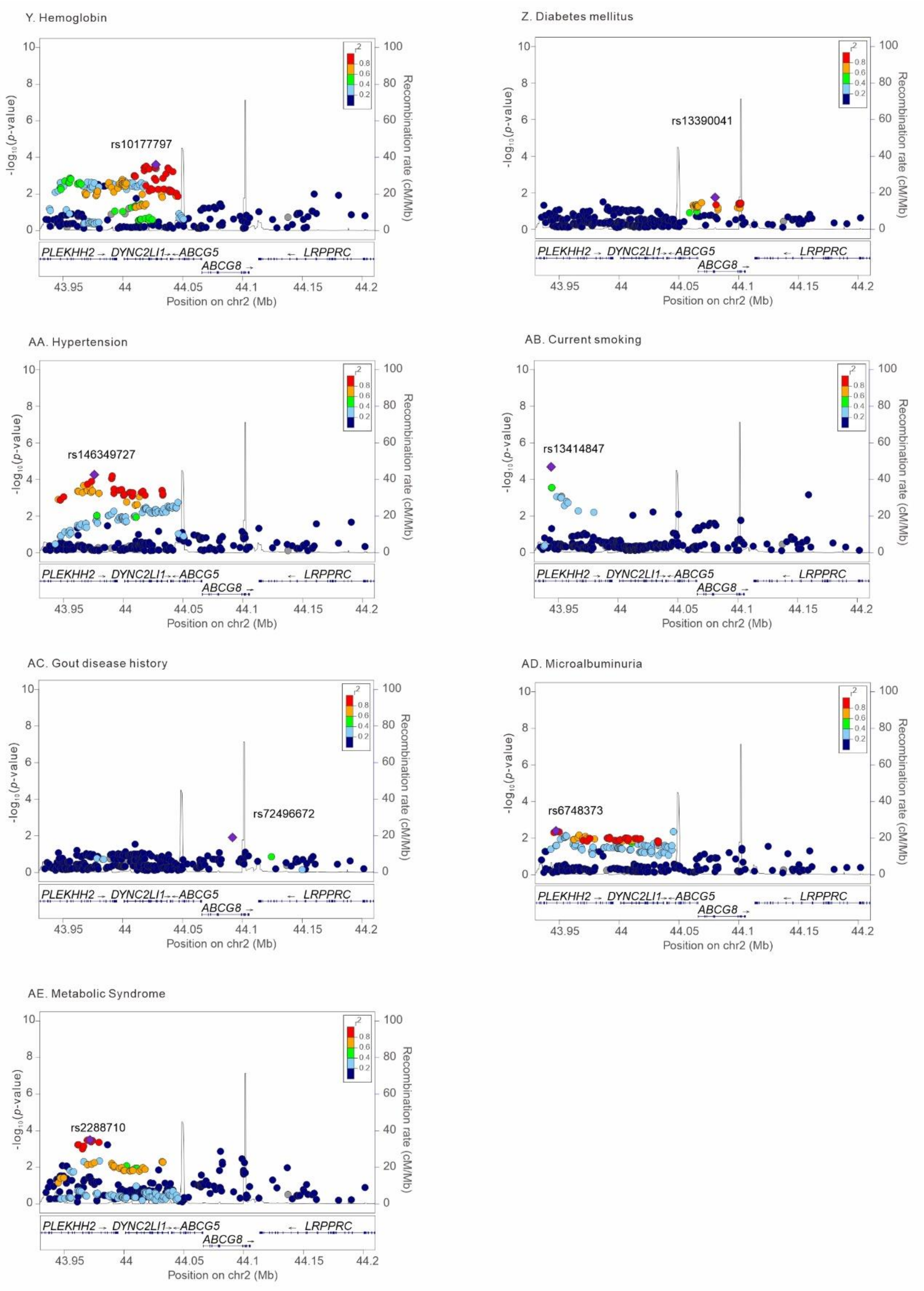
Y. Hemoglobin
rs10177797
Z. Diabetes mellitus
rs13390041
AA. Hypertension
rs146349727
AB. Current smoking
rs13414847
AC. Gout disease history
rs72496672
AD. Microalbuminuria
rs6748373
AE. Metabolic Syndrome
rs2288710
$-\log_{10}(p\text{-value})$
Recombination rate (cM/Mb)
r^2
PLEKHH2
DYNC2LI1
ABCG5
ABCG8
LRPPRC
Position on chr2 (Mb)

Table S8. Association between *ABCG5* rs560839317 genotypes and clinical and laboratory parameters in Taiwan Biobank participants

Clinical and laboratory parameters	beta	SE	*p* value
Anthropology			
Age (years)	-0.1144	0.6186	0.8533
Waist circumference (cm)	0.0845	0.2873	0.7685
Waist-hip ratio	0.0021	0.0030	0.4821
Body mass index (kg/m^2)	0.0095	0.2099	0.9641
Blood Pressure			
Systolic BP† (mmHg)	0.5365	0.8705	0.5377
Diastolic BP† (mmHg)	0.2468	0.5695	0.6648
Mean BP† (mmHg)	0.3435	0.6258	0.5831
Lipid profiles			
Total cholesterol# (mg/dL)	0.0222	0.0046	1.43×10^{-6}
HDL-cholesterol# (mg/dL)	-0.0081	0.0055	0.1376
LDL-cholesterol# (mg/dL)	0.0339	0.0069	8.59×10^{-7}
Triglyceride# (mg/dL)	0.0344	0.0128	0.0073
Non-HDL-cholesterol# (mg/dL)	0.0335	0.0062	6.97×10^{-8}
Remnant cholesterol# (mg/dL)	0.0197	0.0156	0.2067
Glucose metabolism			
Fasting plasma glucose†† (mg/dL)	1.2984	0.8484	0.1259
HbA1c†† (%)	0.0560	0.0338	0.0978
Insulin resistance surrogate markers			
TyG index#, ††	1650.0897	585.3530	0.0048
TyG-BMI#, †† ($\times 10^3$)	43.91035	15.4786	0.0046
TyG-WC#, †† ($\times 10^3$)	148.0184	53.3335	0.0055
Uric acid			
Uric acid††† (mg/dL)	0.0663	0.0631	0.2930
Renal function			
Creatinine (mg/dL)	-0.0145	0.0156	0.3508
eGFR (mL/min/1.73 m^2)	1.9791	1.2241	0.1059
Albuminuria (mg/L)	-0.0244	0.0259	0.3458
Liver function			
AST (U/L)	0.3534	0.6863	0.6066
ALT (U/L)	1.3298	1.1193	0.2348
γGT (U/L)	5.9924	1.7961	0.0008
Serum albumin (g/dL)	-0.0142	0.0127	0.2609
Total bilirubin (mg/dL)	-0.0377	0.0154	0.0145
Hematological parameters			
Leukocyte count (10^3/μL)	-0.1391	1.6657	0.9335
Hematocrit (%)	0.1297	0.1947	0.5053
Platelet count (10^3/μL)	2.2186	3.2985	0.5012
Red blood cell count (10^6/μL)	0.0092	0.0255	0.7169
Hemoglobin (g/dL)	0.0205	0.0705	0.7707
Atherosclerotic risk factors			
Gallstone (%)	-0.0948	0.2853	0.7398
Diabetes mellitus (%)	0.4211	0.1786	0.0184
Hypertension (%)	0.1559	0.1468	0.2883
Current smoking (%)	0.0841	0.1588	0.5961
Gout (%)	0.1503	0.2953	0.6108
Microalbuminuria (%)	-0.1426	0.1923	0.4584
Metabolic syndrome (%)	0.3321	0.1432	0.0204

p value: adjusted for age, sex, BMI, and current smoking; age: adjusted for sex, BMI and current smoking; BMI: adjusted for age, sex and smoking; current smoking: adjusted for age, sex and BMI.
Participant recruitment for analysis is shown in Figure 1 and Supplementary Table 6.
Abbreviation as in Table 1 and Supplementary Table 6.

Table S9. Association of the *ABCG5* rs6720173 genotype with metabolic and hematological phenotypes

Clinical and laboratory parameters	beta	SE	*p* value
Anthropology			
Age (years)	0.1369	0.0731	0.0610
Waist circumference (cm)	-0.0451	0.0339	0.1844
Waist-hip ratio	-0.0002	0.0004	0.5757
Body mass index (kg/m^2)	-0.0295	0.0248	0.2339
Blood Pressure			
Systolic BP† (mmHg)	0.2319	0.1022	0.0233
Diastolic BP† (mmHg)	0.2242	0.0669	0.0008
Mean BP† (mmHg)	0.2268	0.0735	0.0020
Lipid profiles			
Total cholesterol# (mg/dL)	0.0016	0.0005	0.0024
HDL-cholesterol# (mg/dL)	0.0014	0.0006	0.0272
LDL-cholesterol# (mg/dL)	0.0016	0.0008	0.0456
Triglyceride# (mg/dL)	0.0014	0.0015	0.3462
Non-HDL-cholesterol# (mg/dL)	0.0015	0.0007	0.0292
Remnant cholesterol# (mg/dL)	0.0007	0.0018	0.7090
Glucose metabolism			
Fasting plasma glucose†† (mg/dL)	-0.0056	0.1001	0.9556
HbA1c†† (%)	-0.0044	0.0040	0.2712
Insulin resistance surrogate markers			
TyG index#, ††	101.4381	67.2792	0.1316
TyG-BMI#, †† (× 10^3)	3.05413	1.7792	0.0861
TyG-WC#, †† (× 10^3)	10.4686	6.1230	0.0877
Uric acid			
Uric acid††† (mg/dL)	0.0124	0.0074	0.0962
Renal function			
Creatinine (mg/dL)	-0.0003	0.0018	0.8685
eGFR (mL/min/1.73 m^2)	0.0699	0.1447	0.6290
Albuminuria (mg/L)	-0.0054	0.0031	0.0793
Liver function			
AST (U/L)	-0.0952	0.0812	0.2410
ALT (U/L)	-0.0101	0.1324	0.9393
γGT (U/L)	0.1275	0.2130	0.5494
Serum albumin (g/dL)	0.0001	0.0015	0.9617
Total bilirubin (mg/dL)	-0.0027	0.0018	0.1369
Hematological parameters			
Leukocyte count (10^3/μL)	0.3595	0.1968	0.0678
Hematocrit (%)	0.0547	0.0230	0.0174
Platelet count (10^3/μL)	0.2002	0.3897	0.6075
Red blood cell count (10^6/μL)	0.0072	0.0030	0.0171
Hemoglobin (g/dL)	0.0158	0.0083	0.0574
Atherosclerotic risk factors			
Gallstone (%)	0.0389	0.0318	0.2218
Diabetes mellitus (%)	-0.0043	0.0241	0.8570
Hypertension (%)	0.0158	0.0179	0.3770
Current smoking (%)	-0.0064	0.0192	0.7375
Gout (%)	0.0216	0.0361	0.5489
Microalbuminuria (%)	-0.0141	0.0217	0.5172
Metabolic syndrome (%)	-0.0187	0.0178	0.2942

Adjustment condition, participant recruitment and abbreviation as in Supplementary Table 8.

Table S10. Association between *ABCG5* rs199984328 genotypes and clinical and laboratory parameters in Taiwan Biobank participants

Clinical and laboratory parameters	beta	SE	*P* value*
Anthropology			
Age (years)	-0.0674	0.2700	0.8029
Waist circumference (cm)	-0.0086	0.1253	0.9453
Waist-hip ratio	-0.0005	0.0013	0.7182
Body mass index (kg/m^2)	-0.0557	0.0916	0.5427
Blood Pressure			
Systolic BP† (mmHg)	0.1401	0.3783	0.7112
Diastolic BP† (mmHg)	0.5132	0.2474	0.0381
Mean BP† (mmHg)	0.3889	0.2719	0.1527
Lipid profiles			
Total cholesterol# (mg/dL)	0.0116	0.0020	4.92×10^{-9}
HDL-cholesterol# (mg/dL)	-0.0020	0.0024	0.4072
LDL-cholesterol# (mg/dL)	0.0186	0.0030	3.35×10^{-10}
Triglyceride# (mg/dL)	0.0102	0.0055	0.0655
Non-HDL-cholesterol# (mg/dL)	0.0171	0.0027	1.71×10^{-10}
Remnant cholesterol# (mg/dL)	0.0090	0.0068	0.1839
Glucose metabolism			
Fasting plasma glucose†† (mg/dL)	0.4676	0.3695	0.2057
HbA1c†† (%)	0.0173	0.0147	0.2396
Insulin resistance surrogate markers			
TyG index#, ††	584.3825	250.1208	0.0195
TyG-BMI#, †† (× 10^3)	15.5297	6.61444	0.0189
TyG-WC#, †† (× 10^3)	54.1620	22.7899	0.0175
Uric acid			
Uric acid††† (mg/dL)	0.0318	0.0275	0.2476
Renal function			
Creatinine (mg/dL)	0.0118	0.0068	0.0822
eGFR (mL/min/1.73 m^2)	-0.4869	0.5346	0.3624
Albuminuria (mg/L)	-0.0116	0.0113	0.3072
Liver function			
AST (U/L)	0.3702	0.2998	0.2168
ALT (U/L)	1.0852	0.4896	0.0267
γGT (U/L)	-0.0755	0.7875	0.9236
Serum albumin (g/dL)	-0.0067	0.0055	0.2279
Total bilirubin (mg/dL)	-0.0064	0.0067	0.3412
Hematological parameters			
Leukocyte count (10^3/μL)	-0.0006	0.7271	0.9994
Hematocrit (%)	0.1174	0.0850	0.1673
Platelet count (10^3/μL)	0.1415	1.4398	0.9217
Red blood cell count (10^6/μL)	0.0199	0.0111	0.0738
Hemoglobin (g/dL)	0.0106	0.0308	0.7299
Atherosclerotic risk factors			
Gallstone (%)	-0.1375	0.1274	0.2805
Diabetes mellitus (%)	-0.0025	0.0896	0.9774
Hypertension (%)	0.0697	0.0657	0.2886
Current smoking (%)	-0.1778	0.0729	0.0147
Gout (%)	0.0603	0.1312	0.6460
Microalbuminuria (%)	-0.0457	0.0815	0.5748
Metabolic syndrome (%)	0.1970	0.0639	0.0021

Adjustment condition, participant recruitment and abbreviation as in Supplementary Table 8.

Table S11. Association between *ABCG5* rs119480069 genotypes and clinical and laboratory parameters in Taiwan Biobank participants

Clinical and laboratory parameters	beta	SE	*P* value*
Anthropology			
Age (years)	-0.3881	0.4074	0.3407
Waist circumference (cm)	0.0268	0.1892	0.8872
Waist-hip ratio	0.0054	0.0020	0.0056
Body mass index (kg/m^2)	-0.0359	0.1382	0.7947
Blood Pressure			
Systolic BP† (mmHg)	0.6773	0.5677	0.2329
Diastolic BP† (mmHg)	0.1378	0.3713	0.7106
Mean BP† (mmHg)	0.3176	0.4081	0.4364
Lipid profiles			
Total cholesterol# (mg/dL)	0.0261	0.0030	5.02×10^{-18}
HDL-cholesterol# (mg/dL)	-0.0025	0.0036	0.4899
LDL-cholesterol# (mg/dL)	0.0398	0.0045	9.72×10^{-19}
Triglyceride# (mg/dL)	0.0137	0.0084	0.1033
Non-HDL-cholesterol# (mg/dL)	0.0375	0.0041	3.22×10^{-20}
Remnant cholesterol# (mg/dL)	0.0227	0.0103	0.0272
Glucose metabolism			
Fasting plasma glucose†† (mg/dL)	0.1152	0.5613	0.8374
HbA1c†† (%)	-0.0022	0.0224	0.9219
Insulin resistance surrogate markers			
TyG index$^{\#, \dagger\dagger}$	233.2743	382.5686	0.5420
TyG-BMI$^{\#, \dagger\dagger}$ ($\times 10^3$)	9.8945	10.1163	0.3280
TyG-WC$^{\#, \dagger\dagger}$ ($\times 10^3$)	25.6732	34.8567	0.4614
Uric acid			
Uric acid††† (mg/dL)	0.0904	0.0417	0.0303
Renal function			
Creatinine (mg/dL)	-0.0057	0.0103	0.5779
eGFR (mL/min/1.73 m^2)	-0.3193	0.8062	0.6921
Albuminuria (mg/L)	0.0013	0.0171	0.9373
Liver function			
AST (U/L)	1.3172	0.4517	0.0035
ALT (U/L)	1.5330	0.7377	0.0377
γGT (U/L)	2.4041	1.1876	0.0429
Serum albumin (g/dL)	-0.0131	0.0083	0.1164
Total bilirubin (mg/dL)	-0.0025	0.0101	0.8088
Hematological parameters			
Leukocyte count (10^3/μL)	-0.0356	1.0957	0.9741
Hematocrit (%)	0.3565	0.1282	0.0054
Platelet count (10^3/μL)	1.4942	2.1723	0.4916
Red blood cell count (10^6/μL)	-0.0063	0.0168	0.7082
Hemoglobin (g/dL)	0.1250	0.0464	0.0070
Atherosclerotic risk factors			
Gallstone (%)	-0.1560	0.1941	0.4215
Diabetes mellitus (%)	0.0864	0.1309	0.5093
Hypertension (%)	-0.0392	0.1014	0.6987
Current smoking (%)	0.1879	0.1024	0.0665
Gout (%)	0.2635	0.1795	0.1421
Microalbuminuria (%)	-0.0528	0.1225	0.6662
Metabolic syndrome (%)	0.2108	0.0967	0.0292

Adjustment condition, participant recruitment and abbreviation as in Supplementary Table 8.

Table S12. Association between *ABCG8* rs11887534 genotypes and clinical and laboratory parameters in Taiwan Biobank participants

Clinical and laboratory parameters	beta	SE	*P* value*
Anthropology			
Age (years)	0.3301	0.1837	0.0724
Waist circumference (cm)	0.0284	0.0853	0.7393
Waist-hip ratio	-0.0004	0.0009	0.6858
Body mass index (kg/m^2)	-0.0124	0.0623	0.8426
Blood Pressure			
Systolic BP† (mmHg)	0.3215	0.2562	0.2095
Diastolic BP† (mmHg)	0.4000	0.1676	0.0170
Mean BP† (mmHg)	0.3738	0.1842	0.0424
Lipid profiles			
Total cholesterol# (mg/dL)	-0.0008	0.0014	0.5489
HDL-cholesterol# (mg/dL)	-0.0003	0.0016	0.8396
LDL-cholesterol# (mg/dL)	-0.0023	0.0020	0.2401
Triglyceride# (mg/dL)	0.0036	0.0037	0.3334
Non-HDL-cholesterol# (mg/dL)	-0.0015	0.0018	0.4000
Remnant cholesterol# (mg/dL)	0.0045	0.0046	0.3197
Glucose metabolism			
Fasting plasma glucose†† (mg/dL)	0.4600	0.2511	0.0669
HbA1c†† (%)	0.0083	0.0100	0.4070
Insulin resistance surrogate markers			
TyG index$^{\#, \dagger\dagger}$	220.3875	168.6637	0.1913
TyG-BMI$^{\#, \dagger\dagger}$ ($\times 10^3$)	6.2021	4.4600	0.1643
TyG-WC$^{\#, \dagger\dagger}$ ($\times 10^3$)	21.4883	15.3673	0.1620
Uric acid			
Uric acid††† (mg/dL)	-0.0059	0.0187	0.7500
Renal function			
Creatinine (mg/dL)	0.0041	0.0046	0.3747
eGFR (mL/min/1.73 m^2)	-0.0865	0.3636	0.8120
Albuminuria (mg/L)	-1.3956	3.2741	0.6699
Liver function			
AST (U/L)	0.1151	0.2038	0.5721
ALT (U/L)	0.6028	0.3327	0.0700
γGT (U/L)	-0.2668	0.5353	0.6182
Serum albumin (g/dL)	0.0007	0.0038	0.8487
Total bilirubin (mg/dL)	-0.0006	0.0046	0.9034
Hematological parameters			
Leukocyte count (10^3/μL)	-0.0381	0.4939	0.9385
Hematocrit (%)	0.0991	0.0578	0.0867
Platelet count (10^3/μL)	-1.5469	0.9795	0.1143
Red blood cell count (10^6/μL)	0.0029	0.0076	0.7006
Hemoglobin (g/dL)	0.0393	0.0209	0.0601
Atherosclerotic risk factors			
Gallstone (%)	0.3604	0.0694	2.08×10^{-7}
Diabetes mellitus (%)	-0.0710	0.0618	0.2500
Hypertension (%)	0.0054	0.0450	0.9050
Current smoking (%)	-0.0888	0.0494	0.0725
Gout (%)	-0.1014	0.0962	0.2917
Microalbuminuria (%)	0.0825	0.0527	0.1172
Metabolic syndrome (%)	0.0413	0.0443	0.3516

Adjustment condition, participant recruitment and abbreviation as in Supplementary Table 8.

Table S13. Association between *PLEKHH2* rs7596913 genotypes and clinical and laboratory parameters in Taiwan Biobank participants

Clinical and laboratory parameters	beta	SE	*P* value*
Anthropology			
Age (years)	0.1229	0.0568	0.0305
Waist circumference (cm)	-0.0010	0.0264	0.9703
Waist-hip ratio	0.0001	0.0003	0.6601
Body mass index (kg/m^2)	-0.0049	0.0193	0.7993
Blood Pressure			
Systolic BP† (mmHg)	0.3347	0.0794	2.51×10^{-5}
Diastolic BP† (mmHg)	0.2651	0.0520	3.36×10^{-7}
Mean BP† (mmHg)	0.2883	0.0571	4.44×10^{-7}
Lipid profiles			
Total cholesterol# (mg/dL)	0.0010	0.0004	0.0151
HDL-cholesterol# (mg/dL)	-0.0001	0.0005	0.8762
LDL-cholesterol# (mg/dL)	0.0019	0.0006	0.0021
Triglyceride# (mg/dL)	0.0009	0.0012	0.4373
Non-HDL-cholesterol# (mg/dL)	0.0015	0.0006	0.0058
Remnant cholesterol# (mg/dL)	0.0003	0.0014	0.8089
Glucose metabolism			
Fasting plasma glucose†† (mg/dL)	0.0677	0.0778	0.3840
HbA1c†† (%)	-0.0030	0.0031	0.3386
Insulin resistance surrogate markers			
TyG index#, ††	0.0032	0.0029	0.2549
TyG-BMI#, †† ($\times 10^3$)	0.0747	0.0708	0.2914
TyG-WC#, †† ($\times 10^3$)	0.3010	0.3552	0.3966
Uric acid			
Uric acid††† (mg/dL)	0.0059	0.0058	0.3055
Renal function			
Creatinine (mg/dL)	0.0004	0.0014	0.7724
eGFR (mL/min/1.73 m^2)	0.0961	0.1121	0.3912
Albuminuria (mg/L)	-0.0047	0.0024	0.0486
Liver function			
AST (U/L)	-0.0147	0.0622	0.8129
ALT (U/L)	0.1098	0.1024	0.2839
γGT (U/L)	-0.0570	0.1647	0.7295
Serum albumin (g/dL)	0.0005	0.0012	0.6687
Total bilirubin (mg/dL)	0.0001	0.0014	0.9176
Hematological parameters			
Leukocyte count (10^3/μL)	0.1793	0.1530	0.2412
Hematocrit (%)	0.0510	0.0179	0.0043
Platelet count (10^3/μL)	0.4284	0.3029	0.1573
Red blood cell count (10^6/μL)	0.0034	0.0023	0.1431
Hemoglobin (g/dL)	0.0169	0.0065	0.0089
Atherosclerotic risk factors			
Gallstone (%)	0.0229	0.0250	0.3594
Diabetes mellitus (%)	0.0054	0.0187	0.7716
Hypertension (%)	0.0316	0.0139	0.0228
Current smoking (%)	-0.0113	0.0149	0.4492
Gout (%)	-0.0158	0.0284	0.5781
Microalbuminuria (%)	-0.0308	0.0169	0.0680
Metabolic syndrome (%)	0.0136	0.0139	0.3255

Adjustment condition, participant recruitment and abbreviation as in Supplementary Table 8.

Table S14. Association between *PLEKHH2* rs2060173 genotypes and clinical and laboratory parameters in Taiwan Biobank participants

Clinical and laboratory parameters	beta	SE	*P* value*
Anthropology			
Age (years)	0.1162	0.0567	0.0405
Waist circumference (cm)	0.0024	0.0263	0.9278
Waist-hip ratio	0.0001	0.0003	0.5937
Body mass index (kg/m^2)	-0.0093	0.0192	0.6300
Blood Pressure			
Systolic BP† (mmHg)	0.3325	0.0793	2.74×10^{-5}
Diastolic BP† (mmHg)	0.2650	0.0519	3.25×10^{-7}
Mean BP† (mmHg)	0.2875	0.0570	4.58×10^{-7}
Lipid profiles			
Total cholesterol# (mg/dL)	0.0011	0.0004	0.0077
HDL-cholesterol# (mg/dL)	-0.0001	0.0005	0.8992
LDL-cholesterol# (mg/dL)	0.0020	0.0006	0.0012
Triglyceride# (mg/dL)	0.0010	0.0012	0.3865
Non-HDL-cholesterol# (mg/dL)	0.0017	0.0006	0.0028
Remnant cholesterol# (mg/dL)	0.0006	0.0014	0.6844
Glucose metabolism			
Fasting plasma glucose†† (mg/dL)	0.0511	0.0777	0.5107
HbA1c†† (%)	-0.0034	0.0031	0.2750
Insulin resistance surrogate markers			
TyG index#, ††	0.0034	0.0029	0.2371
TyG-BMI#, †† ($\times 10^3$)	0.0767	0.0707	0.2782
TyG-WC#, †† ($\times 10^3$)	0.3351	0.3546	0.3447
Uric acid			
Uric acid††† (mg/dL)	0.0060	0.0060	0.2753
Renal function			
Creatinine (mg/dL)	0.0004	0.0014	0.8031
eGFR (mL/min/1.73 m^2)	0.1098	0.1119	0.3267
Albuminuria (mg/L)	-0.0046	0.0024	0.0510
Liver function			
AST (U/L)	-0.0110	0.0622	0.8601
ALT (U/L)	0.1266	0.1027	0.2178
γGT (U/L)	-0.0379	0.1645	0.8180
Serum albumin (g/dL)	0.0005	0.0012	0.6727
Total bilirubin (mg/dL)	0.0003	0.0014	0.8385
Hematological parameters			
Leukocyte count (10^3/μL)	0.1772	0.1528	0.2463
Hematocrit (%)	0.0506	0.0178	0.0046
Platelet count (10^3/μL)	0.4143	0.3025	0.1708
Red blood cell count (10^6/μL)	0.0034	0.0023	0.1417
Hemoglobin (g/dL)	0.0171	0.0065	0.0082
Atherosclerotic risk factors			
Gallstone (%)	0.0262	0.0249	0.2935
Diabetes mellitus (%)	0.0043	0.0187	0.8197
Hypertension (%)	0.0329	0.0139	0.0176
Current smoking (%)	-0.0125	0.0149	0.4022
Gout (%)	-0.0165	0.0283	0.5600
Microalbuminuria (%)	-0.0299	0.0169	0.0757
Metabolic syndrome (%)	0.0125	0.0138	0.3680

Adjustment condition, participant recruitment and abbreviation as in Supplementary Table 8.
